# Kinetics of Blood Volume and Hemoglobin Mass Adaptation in Sea-Level Newcomers Relocating to Moderate Altitude: A Six-Month Prospective Study

**DOI:** 10.3390/jcm15155833

**Published:** 2026-07-26

**Authors:** Mohammed A. Alshehri, Husain Y. Alkhaldy

**Affiliations:** 1 Central Labs, King Khalid University, P.O. Box 960, Abha 61421, Saudi Arabia; maabdulaziz@kku.edu.sa; 2 Department of Internal Medicine, College of Medicine, King Khalid University, Abha 61421, Saudi Arabia

**Keywords:** altitude acclimatization, hemoglobin mass, blood volume, erythropoiesis, lean body mass, CO rebreathing, plasma volume

## Abstract

**Background/Objectives:** The kinetics of blood volume and hemoglobin mass (Hbmass) adaptation in newcomers to moderate altitude and the time required to reach the hematological phenotype of long-term residents remain poorly defined. We aimed to characterize this trajectory over six months and to compare newcomers with established altitude residents. **Methods:** Thirty-seven healthy male sea-level newcomers were prospectively followed at baseline (within 48 h of arrival), two months, and six months after relocation to Abha, Saudi Arabia (2250 m). Hbmass, blood volume (BV), plasma volume (PV), and red blood cell volume (RBCV) were measured by CO rebreathing; lean body mass (LBM) was assessed by DXA. Six-month values were compared with 107 long-term male altitude residents. Linear mixed-effects models with Holm–Bonferroni correction were used. **Results:** Adaptation followed a biphasic trajectory. At two months, RBCV and Hbmass rose significantly from baseline (RBCV + 186 mL, Hbmass + 65 g; both *p* ≤ 0.005), confirmed by LBM-adjusted analysis. These gains partially regressed by six months, after which no volume parameter differed significantly from baseline. Hemoglobin (Hb) and hematocrit (Hct) remained persistently elevated (+6 g/L, +1.9%; both *p* < 0.001) due to sustained PV contraction. Compared with long-term residents, six-month newcomers had significantly lower LBM-adjusted Hbmass (15.7 vs. 18.0 g/kg), RBCV (48.0 vs. 54.2 mL/kg), and BV (98.9 vs. 109.8 mL/kg; all *p* < 0.001), yet Hb and Hct were not significantly different. **Conclusions:** Moderate altitude acclimatization follows a transient two-month erythropoietic increase, then partial regression. Six months is insufficient to attain the oxygen-carrying capacity of long-term residents’ deficit entirely masked by Hb and Hct. Direct Hbmass measurement normalized to LBM is required for accurate assessment of altitude adaptation.

## 1. Introduction

Blood volume (BV) comprises two compartments—plasma volume (PV) and red blood cell volume (RBCV) that collectively determine oxygen-carrying capacity and hemodynamic performance. In clinical practice, hemoglobin (Hb) and hematocrit (Hct) serve as surrogate markers of red cell status; however, both parameters are concentration-based and therefore susceptible to shifts in plasma volume that may not reflect true changes in red cell mass [1]. A clinician interpreting Hb alone cannot determine whether a given value reflects a genuine change in red cell mass or a compensatory alteration in PV.

Exposure to high altitude drives profound changes in blood volume and its compartments. Within 24 h of arrival, plasma volume contracts in response to hypobaric hypoxia, causing an immediate rise in hemoglobin concentration and hematocrit independent of any change in erythrocyte number [2]. Over subsequent weeks, erythropoietin (EPO) secretion stimulates true expansion of red blood cell volume (RBCV) and hemoglobin mass (Hbmass), further elevating these concentration markers [2,3]. Understanding the kinetics of these changes is essential for interpreting hematological measurements in individuals relocating to altitude.

Cross-sectional studies have established that long-term altitude residents possess substantially greater RBCV and Hbmass than sea-level individuals when values are normalized to lean body mass (LBM) [4,5]. We recently published altitude-specific reference intervals for Hbmass and intravascular volumes in a cohort of 158 moderate-altitude residents at Abha, Saudi Arabia (2250 m), demonstrating that LBM-adjusted Hbmass in male residents (17.9 g/kgᴸᴵᴹ) significantly exceeds that of a sea-level cohort (15.3 g/kgᴸᴵᴹ) [6]. However, that cross-sectional design could not define when or how newcomers reach—or fail to reach—this elevated steady state.

The longitudinal trajectory of hematological adaptation in newcomers to moderate altitude remains poorly characterized. It is unknown whether adaptation follows a linear trajectory, whether an early transient increase occurs, and at what point, if any, the blood volume profile of a newcomer converges with that of long-term residents. Defining this timeline has direct clinical relevance: it determines how long clinicians must wait before applying altitude-specific reference ranges and at which time points concentration-based markers are most likely to be misleading.

This prospective study addressed these questions by following 37 healthy male sea-level newcomers at three time points across six months of residence at moderate altitude (2250 m) and comparing their six-month profiles with those of 107 established altitude residents. We hypothesized that adaptation would follow a biphasic kinetic pattern—an initial transient erythropoietic increase followed by partial regression—and that six months would be insufficient to fully attain the hematological phenotype of long-term dwellers.

## 2. Materials and Methods

### 2.1. Study Design and Ethical Approval

This prospective longitudinal study was conducted at the High Altitude Research Laboratory, King Khalid University, Abha, Saudi Arabia. The study protocol was approved by the Institutional Review Board at King Khalid University (ECM#2023-105) and conducted in accordance with the principles of the Declaration of Helsinki. All participants provided written informed consent after receiving a full explanation of the study and a standardized informational video about the study procedures.

### 2.2. Participants

Forty healthy adult male sea-level newcomers were enrolled. Participants were East African international university students who had been residing at sea level in their home countries for a minimum of two months before their return to Abha. Measurements were obtained within 48 h of their arrival at altitude (baseline) and at two months and six months of continuous altitude residence. Inclusion required age ≥ 18 years and baseline sea-level Hb ≥ 130 g/L. Individuals with known medical conditions, current drug use, or a history of smoking were excluded. Three enrolled participants were excluded during follow-up (measurement errors at one or more time points), yielding a final analytical cohort of 37 participants with complete data across all three visits. A minimum of two months at sea level was required as a washout period; because some participants were returning students who may previously have spent time at 2250 m, any incompletely reversed prior adaptation would tend to raise the early-altitude baseline and thereby make the observed two-month increase a conservative estimate.

For the cross-sectional comparison, the six-month newcomer data were compared with 107 male long-term altitude residents from a simultaneous study by our group using the same measurement protocols and study site [6]. These residents were healthy adult males, lifelong residents of the Abha highlands (Asir region, southwestern Saudi Arabia), who had lived continuously at 2250 m for their entire lives.

### 2.3. Intravascular Volume Measurements

Hemoglobin mass, RBCV, PV, and BV were quantified using a validated, automated carbon monoxide (CO) rebreathing protocol [7,8,9,10,11]. Participants were placed in a recumbent position with an intravenous line secured in the dorsum of the hand. Baseline hemoglobin (g/L), hematocrit (%), and carboxyhemoglobin (%) were measured by blood gas analyzer (Siemens RAPIDPoint 500e, Erlangen, Germany, or Radiometer ABL90, Radiometer Medical ApS, Brønshøj, Denmark). The Detalo Performance device (Detalo Health, Hørsholm, Denmark) administered 99.9% pure CO at 1 mL/kg body weight via a standardized four-minute rebreathing circuit, as previously described in detail by our group [12]. A venous blood sample was collected six minutes post-administration; the rise in carboxyhemoglobin was used to derive Hbmass. RBCV, PV, and BV were then calculated from Hbmass, Hb, and Hct. All primary outputs were device-generated and exported automatically.

### 2.4. Body Composition

Lean body mass was quantified using dual-energy X-ray absorptiometry (DXA; Lunar iDXA, GE Healthcare, Madison, WI, USA). LBM measurements were available for 29 of 37 newcomers at baseline (and corresponding follow-up visits) and for 60 of 107 long-term residents. All intravascular volumes were analyzed as both absolute values and values normalized to body weight (BW) and LBM.

### 2.5. Statistical Analysis

Participant characteristics at baseline are summarized as mean (standard deviation) or median (interquartile range), as appropriate. Longitudinal changes in blood volume parameters were estimated using linear mixed-effects models with a random intercept for each participant and robust (HC3) variance estimation. Models for absolute outcomes were adjusted for age, baseline weight, and height; models for normalized outcomes were adjusted for age and height only. Estimated marginal means and mean differences with 95% confidence intervals (CI) are reported at each timepoint. Multiple comparisons (both two-month versus baseline and six-month versus baseline) were corrected using the Holm–Bonferroni procedure, applied separately within three outcome families: (1) absolute values (BV, PV, RBCV, Hbmass), (2) weight-normalized values, and (3) LBM-normalized values. Hemoglobin and hematocrit were analyzed as unadjusted secondary outcomes. Cross-sectional comparisons between six-month newcomers and long-term residents were performed using linear regression adjusted for age, height, and LBM with robust standard errors. Statistical significance was defined as a two-tailed *p* < 0.05 for all tests. Analyses were performed in StataNow 19.5 (StataCorp, College Station, TX, USA). A fixed seed (12345) was set to ensure full reproducibility of the estimation workflow.

## 3. Results

### 3.1. Participant Characteristics

Baseline characteristics of the 37 newcomers are summarized in Table 1. The cohort had a mean age of 26.5 ± 4.8 years, height of 169.5 ± 5.3 cm, and weight of 68.1 ± 14.1 kg. Mean LBM was 49.9 ± 7.5 kg (available in 29 participants). Baseline Hb was 152 ± 12 g/L with Hct of 46.5 ± 3.6%. Baseline absolute BV was 5008 ± 987 mL, RBCV 2334 ± 513 mL, PV 2674 ± 540 mL, and Hbmass 763 ± 168 g.

### 3.2. Biphasic Kinetics of Adaptation: The Transient Two-Month Increase

Adaptation to altitude followed a non-linear, biphasic trajectory (Table 2). Between baseline and two months, absolute RBCV increased significantly by 186 mL (95% CI: 55 to 317 mL; *p* = 0.005) and Hbmass by 65 g (95% CI: 23 to 108 g; *p* = 0.003). When normalized to LBM, RBCV/LBM increased by 2.9 mL/kg (*p* = 0.015) and Hbmass/LBM by 1.1 g/kg (*p* = 0.007). Weight-normalized values corroborated these findings (RBCV/BW: +2.6 mL/kg, *p* = 0.002; Hbmass/BW: +0.9 g/kg, *p* = 0.001). Absolute BV showed a non-significant upward trend at two months (+192 mL, *p* = 0.119), attributable to the RBCV expansion, as PV did not change materially (+6 mL, *p* = 0.933). Hemoglobin and hematocrit rose significantly from baseline at two months (Hb: +7 g/L, *p* < 0.001; Hct: +1.9%, *p* < 0.001). After Holm-Bonferroni correction within the two-month versus baseline family, the increases in RBCV and Hbmass remained significant.

### 3.3. Six-Month Regression Toward Baseline

Between two and six months, a significant partial regression occurred. Absolute BV decreased by 259 mL (95% CI: −502 to −15 mL; *p* = 0.037) and Hbmass fell by 45 g (95% CI: −86 to −4 g; *p* = 0.032) from their two-month peaks. LBM-adjusted BV declined by 4.8 mL/kg (*p* = 0.026), LBM-adjusted Hbmass by 0.6 g/kg (*p* = 0.046), and LBM-adjusted PV contracted by 3.2 mL/kg (*p* = 0.020). After applying Holm–Bonferroni correction to the six-month versus baseline comparisons, no blood volume parameter remained statistically significant; absolute BV, RBCV, PV, and Hbmass and all normalized counterparts did not differ significantly from baseline by six months.

**Figure 1:**
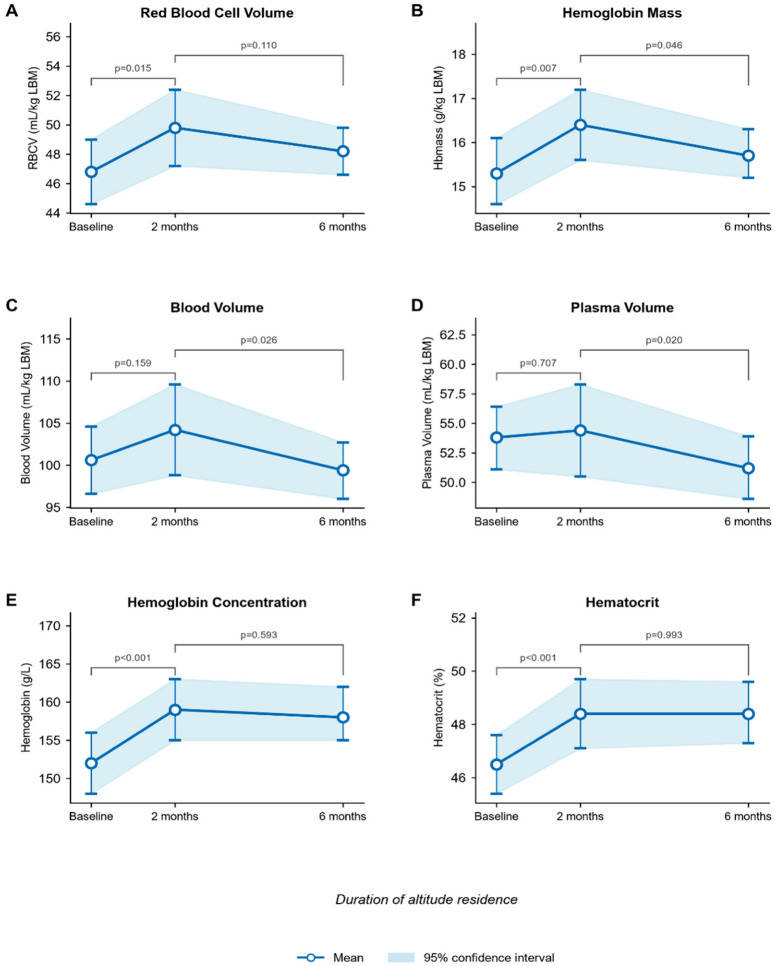
Biphasic kinetics of hematological adaptation in sea-level newcomers relocating to moderate altitude (2250 m) over six months. Panels (**A**–**D**) show LBM-normalized red blood cell volume (RBCV), hemoglobin mass (Hbmass), blood volume (BV), and plasma volume (PV). Panels (**E**,**F**) show hemoglobin concentration (Hb) and hematocrit (Hct). Data points are estimated marginal means from linear mixed-effects models; shaded bands represent 95% confidence intervals. Horizontal brackets indicate *p*-values for pairwise comparisons: left bracket = baseline vs. 2 months; right bracket = 2 months vs. 6 months.

Despite the volumetric regression, Hb and hematocrit remained persistently elevated at six months compared with baseline (Hb: +6 g/L, *p* < 0.001; Hct: +1.9%, *p* < 0.001) and were statistically indistinguishable from their two-month values (Hb: −1 g/L, *p* = 0.593; Hct: 0.0%, *p* = 0.993). This persistent elevation of concentration markers, in the context of returning absolute RBCV and Hbmass, is explained by the progressive contraction of PV observed between two and six months (PV/LBM: −3.2 mL/kg from two to six months, *p* = 0.020; absolute PV: −134 mL, *p* = 0.057) (Figure 1).

### 3.4. Comparison with Long-Term Altitude Residents at Six Months

After six months of altitude residence, newcomers were compared with 107 long-term male altitude residents (Table 3). When expressed as absolute values, residents had significantly greater BV (+561 mL, *p* < 0.001), RBCV (+276 mL, *p* < 0.001), PV (+285 mL, *p* = 0.001), and Hbmass (+110 g, *p* < 0.001). These differences were amplified under LBM-adjusted analysis: residents had 11.4 mL/kgᴸᴵᴹ more BV (*p* < 0.001), 5.8 mL/kgᴸᴵᴹ more RBCV (*p* < 0.001), 5.6 mL/kgᴸᴵᴹ more PV (*p* = 0.003), and 2.30 g/kgᴸᴵᴹ more Hbmass (*p* < 0.001) than six-month newcomers.

**Figure 2:**
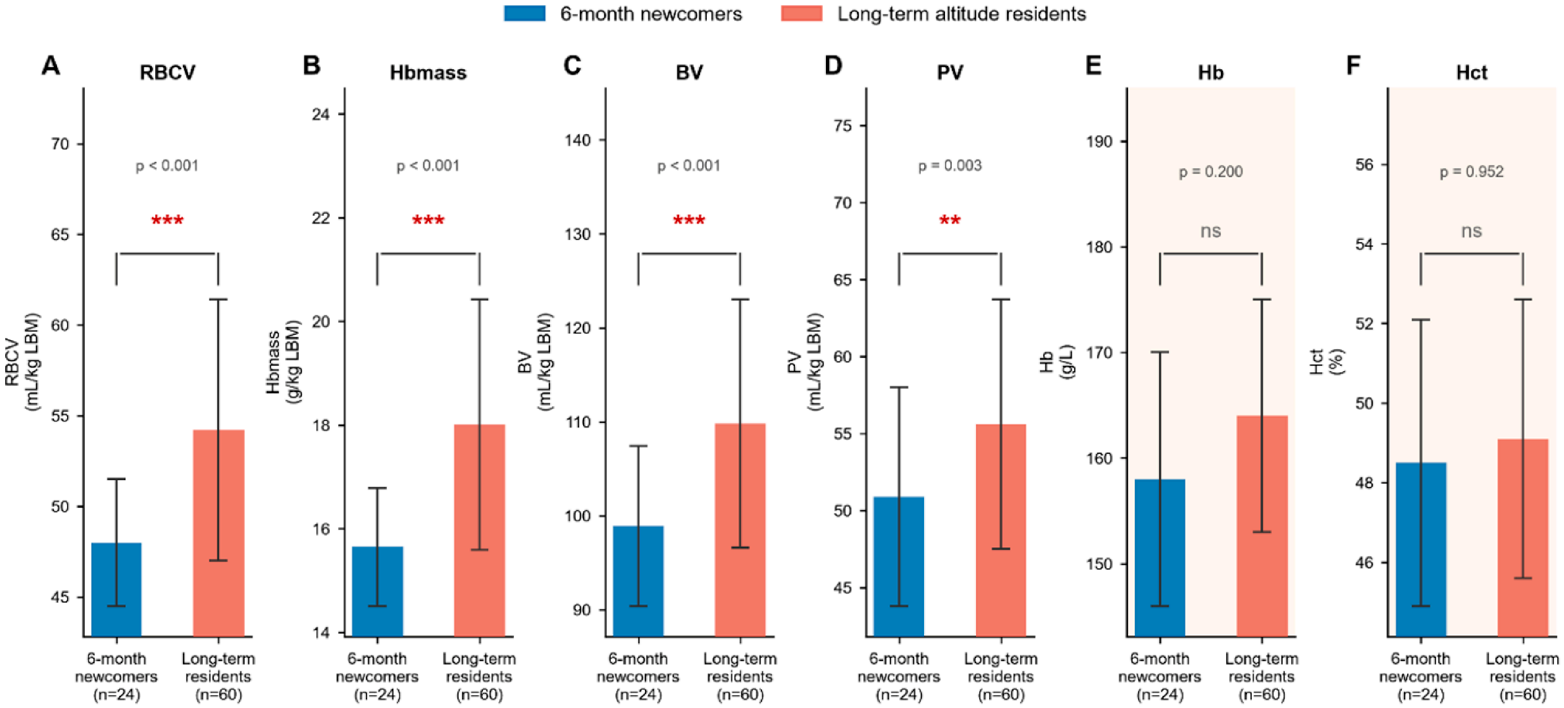
Comparison of LBM-normalized blood volume parameters and concentration markers between 6-month newcomers and long-term altitude residents. Values are mean ± SD. LBM-based analyses: *n* = 24 newcomers, *n* = 60 residents. Shaded panels (**E**,**F**): *p* ≥ 0.05. Panels (**A**–**D**) (RBCV, Hbmass, BV, PV) show LBM-normalized values in which residents significantly exceed 6-month newcomers (all *p* ≤ 0.003). Panels (**E**,**F**) (Hb, Hct) are highlighted in orange to illustrate that concentration markers are statistically indistinguishable between groups (*p* = 0.200 and *p* = 0.952, respectively), despite the marked volumetric differences in panels (**A**–**D**). Values are mean ± SD. LBM-based analyses restricted to participants with available DXA data (*n* = 24 newcomers, *n* = 60 residents). *** *p* < 0.001; ** *p* < 0.01; ns = not significant.

Strikingly, despite these substantial volumetric differences, Hb and Hct did not differ significantly between six-month newcomers and long-term residents (Hb: 158 ± 12 vs. 164 ± 11 g/L, adjusted Δ = +4 g/L, *p* = 0.200; Hct: 48.5 ± 3.6 vs. 49.1 ± 3.5%, adjusted Δ = +0.1%, *p* = 0.952). This finding highlights a fundamental limitation of Hb and Hct: although readily obtained in routine clinical practice, they do not accurately reflect true blood volume or its compartments [13,14]. Recent reviews have further argued that reliance on Hb concentration as a proxy for oxygen-carrying capacity at altitude can obscure meaningful inter-individual and inter-population variation that volumetric measurement alone can reveal [13]. Weight-based normalized comparisons similarly failed to detect significant differences in any blood volume parameter (Figure 2).

## 4. Discussion

This prospective longitudinal study characterized the kinetics of hematological adaptation in healthy male sea-level newcomers over six months of residence at moderate altitude (2250 m). The principal findings are fourfold: (1) adaptation follows a biphasic trajectory, with a significant transient erythropoietic increase at two months followed by partial regression by six months; (2) concentration-based markers (Hb, Hct) remain persistently elevated even as absolute blood volumes regress toward baseline, driven by sustained plasma volume contraction; (3) six months of residence is insufficient to achieve the hematological phenotype of long-term residents; and (4) this shortfall is entirely concealed by Hb and Hct, which are statistically indistinguishable between the two groups despite markedly different true oxygen-carrying capacity.

### 4.1. The Biphasic Pattern: Overshoot and Regression

The transient two-month increase in RBCV and Hbmass reflects the classical erythropoietic response to hypoxia. Upon arrival at altitude, renal EPO secretion increases within hours and peaks within 24–48 h [2]. The resulting expansion of erythropoiesis takes 2–4 weeks to translate into measurable increases in circulating red cell volume and peaks at approximately six to eight weeks [15], consistent with our observed peak at two months. The LBM-adjusted findings confirm that this transient increase represents a genuine expansion of red cell mass rather than a compositional artifact from changes in body weight or fat mass.

The regression between two and six months—where BV and Hbmass declined significantly from their two-month peaks—has been hypothesized to reflect a physiological recalibration in which the erythropoietic stimulus wanes as oxygen delivery is partially restored through other adaptive means. The structural changes proposed (increased capillary density, mitochondrial biogenesis) have not been directly demonstrated at moderate altitude within a six-month timeframe, and this interpretation should be regarded as a working hypothesis requiring prospective mechanistic investigation, and we present it as a hypothesis rather than a demonstrated mechanism. The magnitude of the regression (Hbmass −45 g, BV −259 mL between two and six months) suggests that the response transiently exceeds the eventual steady state before arriving at a stable acclimatized state. Clinicians and researchers measuring blood volumes at the two-month mark should be aware that they are capturing the maximum of a transient peak, not a steady state.

After Holm–Bonferroni correction, no volume parameter was significantly elevated above baseline at six months. This does not mean adaptation has fully resolved—it means that six months of LBM-adjusted values are statistically indistinguishable from baseline sea-level values, yet substantially lower than the values of long-term residents. The newcomers appear to occupy an intermediate, partially acclimatized state that remains distinct from their baseline sea-level phenotype and the hematological profile of long-term residents.

### 4.2. The Hb/Hct Paradox: Concentration Markers as a Misleading Compass

The most clinically important finding of this study is the disconnect between concentration-based and volume-based markers. At six months, Hb and Hct remained elevated above baseline (+6 g/L, +1.9%), yet the underlying RBCV had returned to near-baseline levels. The explanation lies in PV contraction: LBM-adjusted PV continued to decline between two and six months (−3.2 mL/kg, *p* = 0.020), reducing the denominator and thereby maintaining elevated concentration ratios even as the RBCV regressed.

This PV contraction at altitude has been documented previously and appears to be a sustained adaptation rather than a transient response [2,16]. The contraction likely involves mechanisms including altered aldosterone and vasopressin signaling, reduced capillary hydrostatic pressure, and decreased circulating albumin [2,16]. Several alternative mechanisms for the sustained PV contraction merit consideration. Iron depletion is a plausible contributor: the erythropoietic surge at two months creates substantial iron demand that may outstrip supply, potentially suppressing further red cell expansion and contributing to the volumetric regression; iron stores were not assessed in this study. Seasonal variation in ambient temperature and humidity across the three measurement visits may also influence PV through sweat-driven fluid losses independently of altitude adaptation. Hydration status was not standardized across visits. These factors cannot be excluded as partial contributors to the observed PV trajectory.

Whatever its mechanism, its consequence here is that at six months, Hb and Hct paint a false picture of the newcomer’s hematological status: they look elevated relative to sea-level norms (potentially raising concern for polycythemia), yet they are entirely explained by PV contraction rather than true RBCV expansion.

The comparison with long-term residents reveals the full magnitude of this misleading signal. Residents had 2.30 g/kgᴸᴵᴹ more Hbmass (a 15% difference) and 5.8 mL/kgᴸᴵᴹ more RBCV (a 12% difference) than six-month newcomers, yet their Hb and Hct were statistically indistinguishable. Residents achieve this superior oxygen-carrying capacity through substantially expanded red cell mass and plasma volume—both compartments significantly larger than those of 6-month newcomers (Table 3)—such that the hemoglobin concentration ratio is maintained despite markedly greater absolute and LBM-adjusted volumes. A physician reviewing only Hb and Hct would erroneously conclude that a six-month newcomer has “fully adapted”—when in reality the newcomer’s oxygen-carrying capacity is 12–15% lower than that of an established resident. Notably, a 2024 longitudinal study of Sherpas similarly demonstrated marked Hbmass expansion with simultaneous PV contraction at 5400 m, reinforcing that dissociation between volumetric parameters and hemoglobin concentration is a general feature of altitude adaptation, not a population-specific phenomenon [17]. This has important implications for clinical decisions requiring accurate Hbmass assessment, including the evaluation of exercise tolerance, the interpretation of anemia in altitude-dwelling patients, and the differentiation of physiological altitude adaptation from true polycythemia [18,19,20,21,22].

### 4.3. Six Months Is Insufficient: Implications for Adaptation Timelines

Our findings confirm that six months of moderate-altitude residence is insufficient to reach the hematological phenotype of long-term dwellers. This aligns with the broader literature suggesting that full adaptation may require years to decades of continuous altitude residence, potentially involving epigenetic and genomic changes that enhance erythropoietic efficiency and oxygen utilization [14,15,23]. The persistent and substantial gap in LBM-adjusted volumes between newcomers and residents—even after six months—has practical implications for occupational medicine, sports science, and public health in altitude-dwelling communities.

For healthcare workers, soldiers, athletes, and other professionals relocating to altitude postings, the current findings suggest that hematological performance remains below the native-altitude standard for at least six months. From an occupational medicine perspective, this has direct relevance to safe-work guidelines, physical performance standards, and health monitoring protocols for altitude postings—contexts where Hb and Hct are routinely used as primary hematological screens despite their documented inadequacy as volumetric surrogates. Clinicians should not assume that newcomers have “fully adapted” after six months based on normalized Hb or Hct alone; direct measurement of Hbmass provides the only accurate assessment of oxygen-carrying capacity in this context.

For day-to-day clinical practice, these findings translate into a few concrete recommendations. First, a mildly elevated hemoglobin or hematocrit in someone who has recently relocated to moderate altitude should be interpreted as expected plasma-volume contraction rather than polycythemia and does not by itself justify a myeloproliferative work-up. Second, a normal hemoglobin or hematocrit in a newcomer should not be read as evidence of complete adaptation, since true oxygen-carrying capacity remains below the native-resident standard for at least six months. Third, when a decision genuinely depends on oxygen-carrying capacity-unexplained exertional intolerance, distinguishing physiological adaptation from true polycythemia, or interpreting anemia in an altitude dweller-direct hemoglobin-mass measurement by carbon monoxide rebreathing is the accurate test and should be sought where it is available; where it is not, following serial trends over time and assessing iron status is a reasonable alternative to relying on a single concentration value.

These findings carry specific relevance for altitude training strategies, particularly live-high train-low (LHTL) protocols, in which athletes reside at moderate to high altitude to stimulate erythropoiesis while performing high-intensity sessions at lower elevations to maintain training quality. Although direct extrapolation from continuous moderate-altitude residence to structured altitude training camps requires caution, these findings may tentatively inform live-high train-low program design. If the peak erythropoietic response in newcomers occurs at approximately two months, altitude exposures aligned with the ascending phase—roughly six to eight weeks—may theoretically capture the maximal Hbmass stimulus before regression commences. However, LHTL athletes typically undergo intermittent, controlled exposures at variable elevations alongside supervised training loads, making direct comparison with the present continuous-residence cohort speculative. These observations should be treated as hypothesis-generating, and prospective LHTL studies with serial Hbmass measurement are needed to validate this timing principle.

Notably, the two-month newcomers in the present study had Hbmass values approaching those of long-term residents more closely than six-month newcomers, lending further support to the idea that shorter, repeated altitude exposures may be more effective at maximizing erythropoietic gains than prolonged continuous residence.

### 4.4. The Role of LBM Normalization

This study reinforces the importance of LBM as the preferred normalization framework for blood volume assessment at altitude. The transient two-month increase in RBCV and Hbmass was clearly detected by LBM-adjusted metrics (*p* = 0.015 and *p* = 0.007, respectively) but was not apparent from weight-based normalization at six months. Similarly, the gap between newcomers and long-term residents was significant across all LBM-normalized parameters (*p* ≤ 0.003 for all) yet missed by weight-based comparisons (all *p* > 0.077). Since fat tissue is relatively avascular, its inclusion in body weight normalizations dilutes the denominator and attenuates genuine differences in vascular mass [6,24].

The practical implication is straightforward: regardless of altitude, any blood volume assessment should include DXA-derived LBM to ensure accurate interpretation. This is especially important when comparing individuals with different body compositions—a scenario common in altitude medicine, sports physiology, and clinical research.

### 4.5. Limitations

This study has several limitations. First, the cohort comprised exclusively young adult males of East African origin, limiting generalizability to women and other ethnic backgrounds. Female adaptation kinetics may differ given sex-specific differences in EPO sensitivity, iron metabolism, and baseline PV [6]. Second, while the comparison with long-term residents leverages a contemporaneous cohort measured with identical protocols, it is a cross-sectional comparison, and residual confounding cannot be excluded. Third, the study site is a single moderate altitude (2250 m); kinetics at higher elevations may differ substantially. Fourth, LBM data were available for only 29 of 37 newcomers, which may limit power in LBM-normalized analyses. Fifth, the two-month and six-month time points provide snapshots but do not capture the full resolution of the regression phase; additional measurements at 12 months or beyond would further define the trajectory. Sixth, nutritional status, dietary iron intake, and iron stores were not assessed; iron depletion could have contributed to the partial regression in erythropoietic parameters observed between two and six months. Hemoglobin electrophoresis and screening for hemoglobinopathies—including sickle cell trait, which is prevalent in East African populations—were not performed, and the potential influence of hemoglobinopathies on the erythropoietic adaptation trajectory cannot be excluded. Seventh, the biphasic adaptation pattern was characterized at the group mean level; individual trajectories varied, and the pattern should not be assumed to apply uniformly to all newcomers. Eighth, LBM data were available in only 29 of 37 newcomers; while the LBM-normalized results were consistent with absolute and weight-normalized findings, the reduced sample for LBM analyses may have limited statistical power for those comparisons. Ninth, seasonal variation in ambient temperature and humidity across the three measurement visits was not controlled and may have influenced plasma volume measurements independently of altitude adaptation. No a priori sample-size calculation was performed; the sample size was determined by feasibility, and the completed cohort of 37 sits at the larger end of longitudinal hemoglobin-mass studies at altitude. In addition, the baseline was an early-altitude measurement obtained within 48 h of arrival rather than a true sea-level measurement, and because plasma-volume contraction can begin within hours of arrival, this early-altitude baseline may already be mildly contracted—an effect that would make the reported two-month increase a conservative estimate; the exact interval between arrival and measurement was not recorded for each participant. Finally, the newcomers (of East African origin) and the long-term residents (native highland residents) differ in genetic ancestry, which is known to influence hemoglobin regulation at altitude, so adjustment for age, height, and lean body mass cannot fully remove this difference, and the cross-sectional comparison should be interpreted with that in mind.

## 5. Conclusions

At the group level, sea-level newcomers relocating to moderate altitude (2250 m) showed a biphasic hematological pattern: a significant transient erythropoietic increase peaking at two months, followed by a partial regression such that absolute blood volumes were statistically indistinguishable from baseline by six months. Despite this regression, hemoglobin and hematocrit remain persistently elevated, maintained by progressive plasma volume contraction rather than genuine red cell expansion. After six months, newcomers remain significantly below the hematological profile of long-term altitude residents in every LBM-normalized volume parameter, a difference entirely concealed by hemoglobin concentration and hematocrit. These findings demonstrate that concentration-based markers are unreliable guides to the state of altitude acclimatization at any time point examined. Direct measurement of hemoglobin mass and intravascular volumes, normalized to lean body mass, is required for accurate assessment of hematological adaptation and should be considered in any clinical or research context requiring reliable oxygen-carrying capacity estimation at moderate altitude.

## Figures and Tables

**Table 1:** Baseline characteristics of sea-level newcomers (*n* = 37).

Parameter	Mean (SD)
Demographics	
Age (years)	26.5 (4.8)
Height (m)	169.5 (5.3)
Weight (kg)	68.1 (14.1)
Lean Body Mass (kg)	49.9 (7.5)
Hematological Parameters	
Hemoglobin (g/L)	152 (12)
Hematocrit (%)	46.5 (3.6)
Absolute Blood Volumes	
Blood Volume (mL)	5008 (987)
Plasma Volume (mL)	2674 (540)
Red Blood Cell Volume (mL)	2334 (513)
Hemoglobin Mass (g)	763 (168)

Data presented as mean (SD); *n* = 37 for all parameters except lean body mass (LBM), measured in 29 of 37 participants.

**Table 2:** Longitudinal changes in blood volume parameters at baseline, 2 months, and 6 months.

Parameter	Baseline Mean (95% CI)	2 Months Mean (95% CI)	6 Months Mean (95% CI)	Δ2-Mo vs. Base (*p*)	Δ6-Mo vs. 2-Mo (*p*)	Δ6-Mo vs. Base (*p*) ^a^
Absolute Values						
Blood Volume (mL)	5006 (4772–5241)	5198 (4892–5505)	4940 (4743–5137)	+192 (0.119)	−259 (0.037)	−66 (0.489)
Plasma Volume (mL)	2673 (2535–2812)	2680 (2502–2857)	2545 (2418–2673)	+6 (0.933)	−134 (0.057)	−128 (0.050)
RBC Volume (mL)	2332 (2212–2453)	2518 (2356–2681)	2394 (2297–2491)	+186 (0.005)	−124 (0.055)	+62 (0.142)
Hemoglobin Mass (g)	763 (723–802)	828 (775–881)	783 (751–814)	+65 (0.003)	−45 (0.032)	+20 (0.157)
Hemoglobin (g/L)	152 (148–156)	159 (155–163)	158 (155–162)	+7 (<0.001)	−1 (0.593)	+6 (<0.001) ^b^
Hematocrit (%)	46.5 (45.4–47.6)	48.4 (47.1–49.7)	48.4 (47.3–49.6)	+1.9 (<0.001)	0.0 (0.993)	+1.9 (<0.001) ^b^
LBM-Normalized Values						
Blood Volume (mL/kg LBM)	100.6 (96.6–104.6)	104.2 (98.8–109.6)	99.4 (96.0–102.7)	+3.5 (0.159)	−4.8 (0.026)	−1.2 (0.553)
Plasma Volume (mL/kg LBM)	53.8 (51.1–56.4)	54.4 (50.5–58.3)	51.2 (48.6–53.9)	+0.6 (0.707)	−3.2 (0.020)	−2.6 (0.067)
RBC Volume (mL/kg LBM)	46.8 (44.6–49.0)	49.8 (47.2–52.4)	48.2 (46.6–49.8)	+2.9 (0.015)	−1.6 (0.110)	+1.4 (0.155)
Hemoglobin Mass (g/kg LBM)	15.3 (14.6–16.1)	16.4 (15.6–17.2)	15.7 (15.2–16.3)	+1.1 (0.007)	−0.6 (0.046)	+0.4 (0.196)

Data presented as estimated marginal means from linear mixed-effects models. Δ values are model-estimated mean differences (95% CI). ^a^ Holm-Bonferroni correction was applied within each outcome family to both the two-month versus baseline and six-month versus baseline comparisons; unadjusted *p*-values are shown. Holm–Bonferroni correction was applied separately within three outcome families (absolute values, weight-normalized values, and LBM-normalized values); after correction, no primary volume parameter differed significantly from baseline at six months. ^b^—Hb and Hct are secondary outcomes not included in the Holm–Bonferroni correction. LBM = lean body mass; RBC = red blood cell.

**Table 3:** Comparison of Six-Month Newcomers vs. Long-Term Altitude Residents.

Parameter	Newcomers (*n* = 37)	Residents (*n* = 107)	Adj. Δ ^a^	95% CI	*p*-Value
Absolute Values					
Blood Volume (mL)	4838 ± 727	5548 ± 1018	+561	(298 to 825)	<0.001
Plasma Volume (mL)	2489 ± 383	2822 ± 549	+285	(115 to 456)	0.001
RBC Volume (mL)	2349 ± 414	2725 ± 542	+276	(126 to 426)	<0.001
Hemoglobin Mass (g)	766 ± 135	909 ± 181	+110	(60 to 160)	<0.001
Hemoglobin (g/L)	158 ± 12	164 ± 11	+4	(−2 to 10)	0.200
Hematocrit (%)	48.5 ± 3.6	49.1 ± 3.5	+0.1	(−1.7 to 1.8)	0.952
LBM-Normalized Values ^b^					
Blood Volume (mL/kg LBM)	98.9 ± 8.5	109.8 ± 13.2	+11.4	(6.0 to 16.8)	<0.001
Plasma Volume (mL/kg LBM)	50.9 ± 7.1	55.6 ± 8.1	+5.6	(1.9 to 9.2)	0.003
RBC Volume (mL/kg LBM)	48.0 ± 3.5	54.2 ± 7.2	+5.8	(2.9 to 8.7)	<0.001
Hemoglobin Mass (g/kg LBM)	15.65 ± 1.14	18.01 ± 2.41	+2.30	(1.33 to 3.27)	<0.001

Data for newcomers presented as mean ± SD at the six-month visit. Data for residents are from a contemporaneous cross-sectional study (Alkhaldy et al., 2025) [6]. Adjusted Δ = Resident minus Newcomer, adjusted for age, height, and LBM with robust standard errors. ^a^—Positive values indicate higher values in residents. ^b^—LBM-based analyses were restricted to participants with available DXA data (*n* = 24 newcomers, *n* = 60 residents). Abbreviations: RBC, red blood cell; LBM, lean body mass.

## Data Availability

The datasets generated and analyzed during the current study are available from the corresponding author upon reasonable request.
